# Dynamic evolution of lung abnormalities evaluated by quantitative CT techniques in patients with COVID-19 infection

**DOI:** 10.1017/S0950268820001508

**Published:** 2020-07-06

**Authors:** Xinglong Feng, Xuemei Ding, Fuzhou Zhang

**Affiliations:** 1 Department of Anesthesiology, Nanchong Central Hospital, The Second Clinical Medical College, North Sichuan Medical College, Sichuan, Nanchong 637000, China; 2 Department of Radiology, Nanchong Central Hospital, The Second Clinical Medical College, North Sichuan Medical College, Sichuan, Nanchong 637000, China

**Keywords:** Computed tomography, COVID-19, lung, novel coronavirus pneumonia

## Abstract

Chest CT evaluation is often vital to determine patients suspected of COVID-19 pneumonia. The aim of this study was to determine the evolution of lung abnormalities evaluated by quantitative CT techniques in patients with COVID-19 infection from initial diagnosis to recovery. This retrospective study included 16 patients with COVID-19 infection from 30 January 2020 through 11 March 2020. Repeat chest CT examinations were obtained for three or more scans per patient. We measured total volume and mean CT value of lung lesions in each patient per scan, and then calculated the mass, which equals to volume × (CT value + 1000). Dynamic evolution of chest CT imaging as a function of time was fitted by non-linear regression model in terms of mass, volume and CT value, respectively. According to the fitting curves, we redefined the evolution of lung abnormalities: progressive stage (0–5 days), infection emerged and rapidly aggravated; peak stage (5–15 days), the greatest severity at approximate 7–8 days after onset; and absorption stage (15–30 days), the lesions slowly and gradually resolved.

## Introduction

Coronavirus disease-19 (COVID-19), formerly known as 2019 novel coronavirus (2019-nCoV), has been declared to be a global health emergency by the World Health Organization [1, 2]. With the gradual recognition of COVID-19 infection, criteria and guidelines are being steadily updated [3]. Chest CT evaluation is often vital to determine patients suspected of COVID-19 pneumonia, and the hallmark of thoracic imaging are bilateral and peripheral ground-glass opacities including lung interstitial inflammation and extensive consolidation [4, 5]. A recently published literature has revealed the time course of lung changes on chest CT during recovery from COVID-19 pneumonia [6]. However, the semi-quantitative CT score used may be limited by accuracy and sensitivity. The purpose of this study was to determine dynamic evolution of lung abnormalities evaluated by quantitative CT technique in patients with COVID-19 infection.

## Materials and methods

The Ethics Committee of our hospital approved this study, which was in accordance with Helsinki Declaration. Informed consent for this retrospective study was waived.

### Patients

We retrospectively reviewed the medical records of patients with COVID-19 pneumonia diagnosed by the criteria from National Health Commission of the People's Republic of China, including epidemiological history, clinical manifestations, and especially with regard to laboratory diagnosis, the positive real-time fluorescence polymerase chain reaction (RT-PCR) of COVID-19 in throat swabs or sputum. We excluded patients if the number of chest CT examinations per patient was less than three, or their chest CT findings were consistently negative.

### Chest CT evaluation

An appropriate multi-detector CT scanner (SOMATOM Definition AS, Siemens Healthineers, Germany) for patients suspected of COVID-19 infection was used for chest CT acquisitions with tube voltage of 120 kVp and automatic tube current modulation. The raw images were reconstructed with a matrix of 512 × 512 and thickness of 1.5 mm.

Lung abnormalities on chest CT images were measured in terms of total volume (cm^3^) and mean CT value (window level, −600 HU; window width, 1600 HU) using digital database system (Multi Workstation MMWP VE4.0, Siemens) by two radiologists (F.Z. and X.D., who had 10 years and 8 years of experience in thoracic radiology, respectively).

### Statistical analysis

The SPSS software (PASW Statistics 18, SPSS Inc., Chicago, IL, USA) was used for statistical analysis, and *p*-value of less than 0.05 was considered statistically significant. We estimated the inter-observer agreement using Bland−Altman plots for mass, which is equal to volume × (CT value + 1000) [7, 8].

First, we calculated the ratio of each check point to the initial point in each patient in terms of mass and volume, and then standardised each ratio with *z*-score transformation. These standardised ratios of mass and volume, as well as mean CT value of lung abnormalities, as a function of time was quantitatively assessed using non-linear regression module, respectively.

## Results

A total of 16 patients (nine men and seven women, mean age of 43.6 years ±15.5) with COVID-19 pneumonia were included (Table 1). Fever (31.3%) and cough (37.5%) were of the most prevalent symptoms, and three patients were diagnosed with severe pneumonia. Laboratory results were often normal. The severity of clinical symptoms was not significantly correlated with the severity of lung abnormalities (*P* = 0.099).

**Table 1.** Characteristics of the included patients with COVID-19 pneumonia

Characteristics	
Age (years)	43.6 ± 15.5 (10–67)
Gender (male:female)	9:7
Initial symptoms	
Fever	5 (31.3%)
Cough	6 (37.5%)
Diarrhoea	4 (25%)
Myalgia	3 (18.8%)
Symptomless	4 (25%)
Laboratory examinations	
White blood cell count (G/L)	5.9 ± 2.0 (2.6–9.1)
Lymphocyte percentage (%)	20.4 ± 12.3 (0.2–46.3)
C-reactive protein (mg/l)	19.7 ± 25.6 (0.5–94.6)
Calcitonin (ng/ml)	0.07 ± 0.05 (0.02–0.25)
Chest CT findings	
Bilateral	12 (75%)
Multifocal	14 (87.5%)
Subpleural	15 (93.8%)
Ground-glass opacity	12 (75%)
**Interstitial thickening**	**9 (56.3%)**

The initial chest CT scans were conducted 1.0 ± 0.8 days (range: 0–3 days) before standard regimen treating COVID-19. Each patient underwent 5.2 ± 1.0 chest CT scans (range: 3–7 scans) with a duration of 27.5 ± 5.4 days (range: 19–34 days).

### Inter-observer agreement

**Fig. 1.**
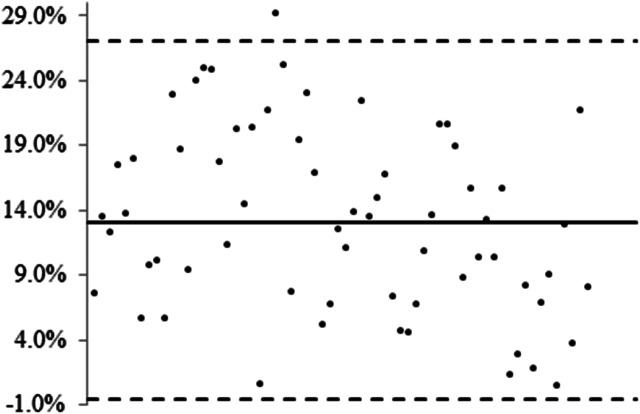
Inter-observer agreement by Bland−Altman plots. The mean of measurement differences for mass is 13.1% ± 7.0% with 95% limits of agreement from −0.6% to 26.8%. The ratio of standard deviation to mean of measurement differences is used to avoid excessive differences in absolute values. The positive measurement differences indicate one observer tends to measure the lung abnormalities more than the other.

The inter-observer measurement variability of mass was 13.1% ± 7.0 with 95% limits of agreement from −0.6% to 26.8% (Fig. 1). Here, we calculated the ratio of standard deviation to mean of measurement differences to avoid excessive differences in absolute values. The measurement variability of mass by Bland−Altman plots indicated that inter-observer agreement was acceptable, although one observer tended to measure the lung abnormalities more than the other (measurement differences were positive).

### Lung abnormalities as function of time

Ground-glass opacity and interlobular/intralobular septal thickening were the most frequent chest CT manifestations, and lung abnormalities were of bilateral, peripheral and multifocal features. The mean mass was 60 425 ± 98 545 at initial diagnosis, and the absorption rate of lung abnormalities was independent of initial mass (*P* = 0.279).

**Fig. 2.**
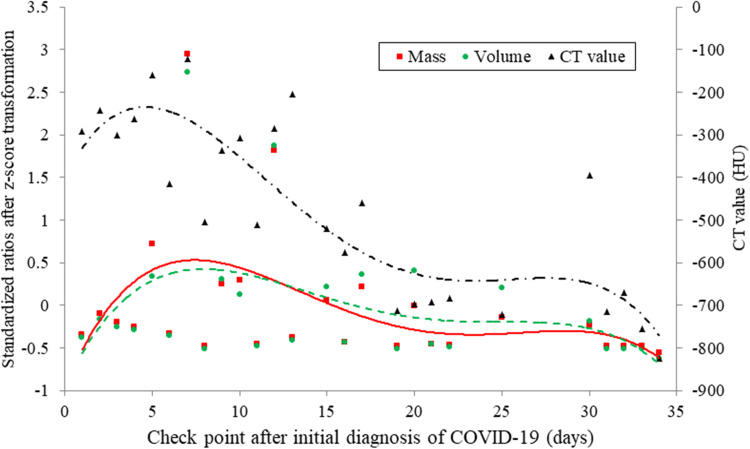
The fitting curves for lung abnormalities evaluated by quantitative CT techniques as a function of time. The evolution of chest CT imaging is defined as progressive stage (0–5 days), peak stage (5–15 days) and absorption stage (15–30 days) by multiple CT quantitative techniques, including mass (*y* = −0.00002×^4^ + 0.0019×^3^ − 0.0519×^2^ + 0.4889*x* − 0.9626, *R*^2^ = 0.2283), volume (*y* = −0.00002×^4^ + 0.0017×^3^ − 0.0441×^2^ + 0.429*x* − 0.9479, *R*^2^ = 0.1878) and CT value (*y* = −0.0068×^4^ + 0.5043×^3^ − 11.976×^2^ + 80.978*x* − 400.18, *R*^2^ = 0.7143).

In patients with COVID-19 infection from onset to recovery, three stages of evolution on chest CT were defined (Fig. 2): progressive stage (0–5 days), infection emerged and rapidly aggravated; peak stage (5–15 days), the greatest severity showed approximately 7–8 days from onset; absorption stage (15–30 days), the lesions were gradually absorbed within 2 weeks. This dynamic evolution was identified by quantitative CT techniques in terms of mass (*y* = −0.00002×^4^ + 0.0019×^3^ − 0.0519×^2^ + 0.4889*x* − 0.9626, *R*^2^ = 0.2283), volume (*y* = −0.00002×^4^ + 0.0017×^3^ − 0.0441×^2^ + 0.429*x* − 0.9479, *R*^2^ = 0.1878) and CT value (*y* = −0.0068×^4^ + 0.5043×^3^ − 11.976×^2^ + 80.978*x* − 400.18, *R*^2^ = 0.7143), respectively.

## Discussion

In the current study of COVID-19 pneumonia from initial diagnosis until recovery, we identified three stages of dynamic evolution of lung abnormalities on CT imaging by using quantitative CT techniques including CT value, volume and mass of lesions: progressive stage (0–5 days), peak stage (5–15 days) and absorption stage (15–30 days). Our result confirmed the conclusion conducted by qualitative and semi-quantitative CT techniques [4, 6].

Bernheim *et al*. [4] qualitatively reviewed chest CT findings in relationship to the time between symptom onset and the initial CT scans in 121 symptomatic patients with COVID-19, and defined imaging hallmarks for early stage (0–2 days), intermediate stage (3–5 days) and late stage (6–12 days). Pan *et al*. [6] semi-quantitatively evaluated the time course of lung changes on chest CT during recovery from COVID-19 pneumonia by using total CT score, which was the sum of lung involvement (five lobes, score 1–5 for each lobe). They defined four stages of evolution on chest CT in patients recovering from COVID-19 infection: early stage (0–4 days), progressive stage (5–8 days), peak stage (10–13 days) and absorption stage (≥14 days). Actually, the boundary between early stage and progressive stage is very vague whether according to their fitting curve or ours. Therefore, we redefined the stages of chest CT evolution: stages 1 and 2 were merged into progressive stage (0–5 days), peak stage was extended to 5–15 days, but absorption stage remained.

Our study has several limitations. First, the small sample size may weaken the details of dynamic evolution of lung abnormalities, although overall trend may remain unchanged. Larger numbers, therefore, would be needed to determine if the timing of lung abnormalities evolution remains as shown in the current study. Second, we do investigate correlations of chest CT findings with clinical manifestations at initial diagnosis. The dynamic correlations from initial diagnosis until patient recovery, however, should be investigated in the future. Third, the retrospective nature and small sample size of this study may introduce observe bias, which likely influences the determination of dynamic features of chest CT findings.

In summary, the evolution of lung abnormalities on CT imaging was redefined as progressive stage (0–5 days), peak stage (5–15 days) and absorption stage (15–30 days) by multiple CT quantitative techniques. The current results of dynamic evolution may be crucial to properly understand the imaging features of COVID-19 pneumonia to improve patient management and determine patient outcomes. However, larger sample size would be needed to determine the time course of chest CT findings.

## Data Availability

The data that support the findings of this study are available from the corresponding author, F.Z., upon reasonable request.
